# Postoperative analgesia in patients undergoing thoracotomy: A comparison between total intravenous anesthesia and inhalation anesthesia

**DOI:** 10.12669/pjms.40.10.9907

**Published:** 2024-11

**Authors:** Joo-Yong Lee, Soon-Taek Jeong, Ji-Hye Hwang, Sang Hi Park

**Affiliations:** 1 Joo-Yong Lee, Department of Anesthesiology and Pain Medicine, Chungbuk National University Hospital, Korea; 2 Soon-Taek Jeong Department of Anesthesiology and Pain Medicine, Chungbuk National University College of Medicine, Cheongju, Korea, Charm Pain Clinic, Cheongju, Korea; 3 Ji-Hye Hwang, Department of Anesthesiology and Pain Medicine, Chungbuk National University Hospital, Korea; 4 Sang Hi Park, Department of Anesthesiology and Pain Medicine, Chungbuk National University Hospital, Korea

**Keywords:** General Anesthesia, Inhalation Anesthesia, Intravenous Anesthesia, Post-operative Pain

## Abstract

**Objective:**

Propofol is more effective than inhalational anesthesia; however, the results for the management of acute pain remain controversial. Therefore, this study aimed to determine the incidence of acute pain after inhalation anesthesia and total intravenous anesthesia among patients who underwent thoracotomy at our hospital.

**Methods:**

We conducted a single center retrospective observational study using data from electronic medical records. Sixty patients aged ≥20 years with American Society of Anesthesiologists physical status class I or II who underwent regular and emergency thoracotomy between January 1, 2016, and January 1, 2020, at Chungbuk National University Hospital were included in this study. The anesthesia and postoperative pain records of those who received total intravenous anesthesia (n=30) and inhalation anesthesia (n=30) were retrospectively reviewed. The pain score on the numeric rating scale (NRS) was evaluated at 2, 8, 24, and 30 hours postoperatively.

**Results:**

The average NRS score of patients who received total intravenous anesthesia was lesser than that of those who received inhalational anesthesia. Moreover, the difference in the NRS scores at eight hours postoperatively was statistically significant (P <0.05). Patients who received inhalational anesthesia had a higher pain score and experienced more severe pain than those who received intravenous anesthesia.

**Conclusions:**

Total intravenous anesthesia with propofol-remifentanil provided better analgesia for acute postoperative pain in patients who underwent thoracotomy than inhalational anesthesia, suggesting it may be considered the combination of choice for thoracic surgery.

***Abbreviations:***

**ASA:** American Society of Anesthesiologists, **CRTS**: Chronic post-thoracotomy pain syndrome, **GABA:** Gamma-aminobutyric acid, **IL:** Interleukin, **NRS:** numeric rating scale.

## INTRODUCTION

Patients undergoing lung surgery experience severe pain more frequently than those undergoing abdominal or upper and lower extremity surgery. Severe postoperative pain impairs lung functions, leading to difficulty in deep breathing, ineffective coughing, and a prolonged postoperative recovery period.^1^,^2^ Recent advances in thoracoscopy have minimized the number of surgical incisions, thereby resulting in a reduction in postoperative pain.

However, patients undergoing lobectomy or pleural adhesion removal are classified as critically ill patients and require strong narcotic analgesics in the recovery room or intensive care unit. Furthermore, chronic surgical site pain persists for a longer duration postoperatively, necessitating pain control during and after the surgery. Chronic post-thoracotomy pain syndrome (CPTS) is defined as pain that persists for at least two months postoperatively due to unknown causes. The incidence of CPTS ranges from 20–80% among patients undergoing thoracotomy and 24–47% among those undergoing video-assisted thoracoscopic surgery.

It has been reported that the choice of anesthesia affects the incidence of postoperative pain. Propofol inhibits the nociceptive transmission of neurons by acting on the gamma-aminobutyric acid (GABA) and glycine receptor receptors. Therefore, it is more effective than inhalational anesthesia in reducing the pain associated with CPTS.^3^ However, the differences between the results of inhalational anesthesia and those of total intravenous anesthesia in the management of acute pain remain controversial.^4^ Therefore, this study aimed to identify an effective anesthetic for patients undergoing thoracic surgery by comparing the incidence of acute pain after inhalational anesthesia and total intravenous anesthesia among patients who underwent thoracotomy.

## METHODS

We conducted a single-center retrospective observational study using data from electronic medical records. This study included patients aged ≥20 years with American Society of Anesthesiologists (ASA) physical status class I or II who underwent regular and emergency thoracotomy between January 1, 2016, and January 1, 2020, at Chungbuk National University Hospital. The anesthesia and postoperative pain records of 60 patients who received total intravenous anesthesia (n=30) and inhalation anesthesia (n=30) were retrospectively reviewed.

### Ethical Approval:

This study was approved by the Institutional Review Board of the Chungbuk National University Hospital (IRB no. 2022-11-020-001, dated: December 29, 2022). Obtaining informed consent was waived due to the retrospective nature of the study.

Patients did not receive any sedative premedication. Routine monitoring procedures, including noninvasive blood pressure measurement, electrocardiography, and pulse oximetry, were conducted at the operating bed in the operating room. In addition, bispectral index (BIS complete 2-channel monitor, Covidien) was applied on the patients’ foreheads to monitor the depth of anesthesia. In the inhalation group, anesthesia was induced using propofol 1–2 mg/kg and neuromuscular blockade was achieved using rocuronium 0.8 mg/kg. Anesthesia was maintained using sevoflurane (O_2_/air mixture: FiO_2_, 50%) and remifentanil target-controlled infusion (effect-site concentration [Ce] set to 4.0 ng/ml). In the total intravenous anesthesia group, general anesthesia was induced using propofol and remifentanil (Ultiva Inj., GlaxoSmithKline Manufacturing S.p.A., target-controlled infusion mode, effect site concentration 3.0 ng/ml in the Minto model). The only difference between the two groups was whether sevoflurane or propofol was used to maintain anesthesia during surgery; otherwise, the anesthesia methods were the same. Both groups received remifentanil for intraoperative pain control, within the range of 2–6 ng/ml, depending on the depth of anesthesia. Remifentanil was discontinued after surgery.

Data regarding the patients’ age, height, weight, and ASA class were collected. In addition, the data regarding the operative technique, duration of anesthesia, and frequency of administering postoperative anti-inflammatory analgesic or narcotic analgesic were compared. The severity of pain, quantified using the numerical rating scale (NRS), was evaluated at 2, 8, 24, and 30 hours postoperatively.

### Statistical analysis:

In a previous pilot experiment, 81% of patients with an NRS score of four or higher at two hours after surgery had received inhalation anesthesia and 39% had received total intravenous anesthesia. Based on these results, we measured a statistical power of 80% (α = 0.05) using the G-power test, and considering a dropout rate of 10%, the final sample size was set to 30 patients per group. Student’s t-test was used for the comparison of continuous data between the inhalational anesthesia and total intravenous anesthesia groups. Normality was confirmed using the Shapiro–Wilk test and the chi-square test was used for categorical data. Descriptive statistics are expressed as the mean ± standard deviation, median (min-max), frequency distribution, and percentage. All statistical analyses were performed using the SPSS for Windows version 27.0 (IBM Corp., USA). Statistical significance was set at P < 0.05.

## RESULTS

No statistically significant differences in sex, age, height, or weight were observed between the two groups. Furthermore, no statistically significant differences were observed in the ASA grade and the surgery or anesthesia time (Table-I).

**Table-I:** Demographic Data.

	Inhalation anesthesia (n=30)	TIVA (n=30)
Sex ratio (M/F)	21/9	20/10
Age (yr)	58.33 ± 15.98	62.13 ± 14.71
Height (cm)	161.51 ± 9.18	161.79 ± 10.40
Weight (kg)	62.49 ± 13.63	64.95 ± 11.57
ASA (I/II)	9/21	8/22
Anesthetic time (min)	145.50 ± 87.73	169.67 ± 84.49
Operative time (min)	109.33 ± 84.12	131.00 ± 78.28

Values represent the number of patients or the mean±standard deviation. There were no significant differences between the two groups. TIVA, Total intravenous anesthesia; ASA, The American Society of Anesthesiologists Physical Grades.

The pain scores were compared between two hours and 30 hours postoperatively, when the effect of general anesthesia had waned. Although no statistically significant difference was observed, the overall postoperative pain score was lower in the total intravenous anesthesia group than in the inhalational anesthesia group.

**Fig.1:**
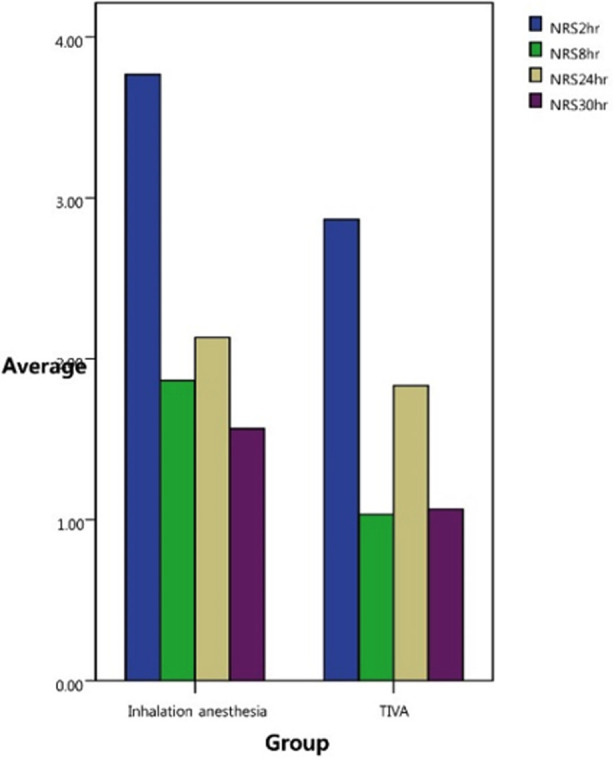
Numerical rating scale (NRS) scores for pain over time. NRS scores are expressed as a median. NRS scores for postoperative pain after 8 hours were significantly different between the two groups (P <0.05). NRS2hr, numeric rating scale 2 hours postoperatively; TIVA, total intravenous anesthesia.

**Fig.2:**
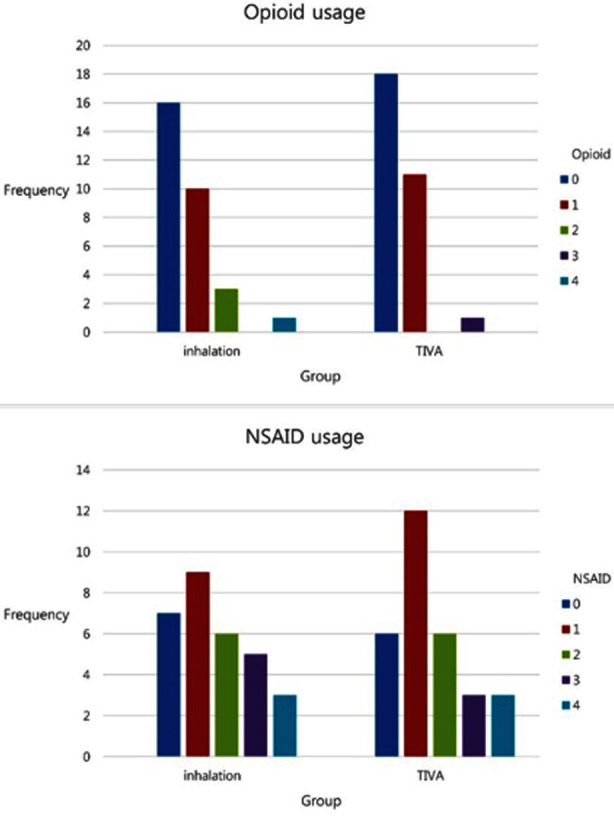
Postoperative analgesia. TIVA, Total intravenous anesthesia; NSAID, Non-steroidal anti-inflammatory drug.

The average NRS scores of patients who received inhalational anesthesia were higher than those of patients who received total intravenous anesthesia. In particular, the NRS score at eight hours postoperatively was significantly associated with inhalational anesthesia (P <0.05). Patients who received inhalational anesthesia had a significantly higher pain score (Fig. 1). Although the total intravenous anesthesia group required a higher amount of NSAIDs and opioids postoperatively, the difference was not statistically significant (Fig.2). Consequently, their overall postoperative pain score was lower than expected.

## DISCUSSION

In this study, the pain score was confirmed retrospectively by referring to the postoperative pain records of patients who underwent thoracotomy. Patients who received total intravenous anesthesia had a significantly lower pain score than those who received inhalational anesthesia. However, there was no difference in the frequency of postoperative analgesic use. This result may be attributed to the use of a low-concentration narcotic analgesic (1.5 mL/hr.) in the patients who received total intravenous anesthesia. Conversely, the difference in the amount of postoperative narcotic analgesic used between patients who received propofol and inhalational anesthesia was insignificant.

The effect of total intravenous anesthesia on postoperative pain has been studied for several surgeries.^5^,^6^ However, studies on thoracic surgery are limited, and its effectiveness remains controversial.^7^ In a study on patients who underwent total laparoscopic hysterectomy, the effects of propofol and sevoflurane on postoperative pain were not significantly different for acute pain, but the propofol group showed lower pain results after 72 hours and at seven days.^8^ In a study on patients who underwent thyroidectomy, pain intensity was higher in the group that received propofol than in the inhalation anesthesia group.^9^ In addition, a randomized controlled study conducted by Wong et al. showed no difference in pain intensity between the total intravenous anesthesia and inhalation anesthesia groups in patients who underwent colorectal surgery.^10^

Many studies comparing propofol and inhalation anesthesia, as discussed above, have shown differences in postoperative analgesic effects, and other studies have shown conflicting results. The largest meta-analysis and systematic review of 39 randomized controlled trials and over 4,500 patients found no differences in postoperative analgesia and opioid consumption between patients who received propofol-based total intravenous anesthesia and those who received volatile anesthetics.^11^ This suggests that the meta-analysis has a limitation of high heterogeneity owing to varying types of surgeries. Therefore, individual studies of patients undergoing thoracic surgery are needed to identify effective anesthetics for these patients. This study is meaningful as it compared the incidence of acute pain following inhalation anesthesia and total intravenous anesthesia in patients undergoing thoracotomy and confirmed the acute analgesic effect of propofol. It also highlights the effectiveness of total intravenous anesthesia in managing short-term acute pain following lung surgery.

Propofol is primarily used for the induction and maintenance of anesthesia. It is commonly used as an anesthetic during endoscopy or outpatient surgery as it is not associated with the incidence of a hangover during the recovery period, unlike other intravenous anesthetics.^12^ Remifentanil, used in conjunction, is an ultra-short-acting opioid analgesic. It is commonly used during general anesthesia because it provides not only antihypertensive but also analgesic effects.^13^

Propofol induces general anesthesia by promoting GABA receptor-inhibitory neurotransmission; however, its exact mechanism of action remains unclear.^14^ In addition, propofol inhibits pain transmission by inhibiting pro-inflammatory cytokines, such as interleukin (IL)-β, IL-6, and tumor necrosis factor-α. N-methyl-D-aspartate plays a crucial role in the transmission and maintenance of pain signals. A previous study has reported that it also modulates the receptor.^14^ Thus, intravenous propofol and remifentanil were predicted to provide superior postoperative pain relief. A previous study on surgical patients reported that patients receiving total intravenous anesthesia with propofol experienced lesser pain than those receiving inhalational anesthesia.^3^,^15^

However, the opposite result has also been reported. Pokkinen et al. (2014) compared the degree of postoperative pain and number of opioids consumed in patients who received propofol and sevoflurane. Unlike our study, they found no significant effect of the anesthetic on the degree of postoperative pain. On the other hand, similar to our findings, the number of opioids required postoperatively was higher in the propofol group, although this difference was not statistically significant.^16^ Song et al. found no significant difference in acute pain following intravenous and inhalation anesthesia, but the group using propofol and remifentanil had significantly reduced post-thoracotomy pain at three and six months. Moreover, long-term pain relief was documented in patients who received total intravenous anesthesia.^4^ Since many studies have correlated the incidence of acute postoperative pain with chronic pain,^17^,^18^ the management of acute postoperative pain by administering the most appropriate anesthetic is essential.

### Limitations:

This study is a retrospective study of a small number of patients in a single center. Therefore, further prospective, randomized studies with large sample sizes must be conducted in the future to validate these results. The degree of pain varied among patients undergoing thoracotomy depending on the scope and time of the surgery. Moreover, the patient population can be further distinguished, such as those undergoing lobectomy or video-assisted thoracotomy.

## CONCLUSION

Total intravenous anesthesia with propofol-remifentanil was more effective than inhalation anesthesia in terms of providing analgesia for acute postoperative pain among patients who underwent thoracotomy.

### Authors Contribution:

**JL** and **SP:** Conceived and designed the study.

**JL**, **SJ**, **JH**, and **SP:** Collected the data and performed the analysis.

**JL** and **SP:** Was involved in the writing of the manuscript and are responsible for the accuracy and integrity of the study.

## References

[1] Muehling BM, Halter GL, Schelzig H, Meierhenrich R, Steffen P, Sunder-Plassmann L (2008). Reduction of postoperative pulmonary complications after lung surgery using a fast track clinical pathway. Eur J Cardiothorac Surg.

[2] Kehlet H, Dahl JB (2003). Anaesthesia, surgery, and challenges in postoperative recovery. Lancet.

[3] Cheng SS, Yeh J, Flood P (2008). Anesthesia matters:patients anesthetized with propofol have less postoperative pain than those anesthetized with isoflurane. Anesth Analg.

[4] Song JG, Shin JW, Lee EH, Choi DK, Bang JY, Chin JH (2012). Incidence of post-thoracotomy pain:a comparison between total intravenous anaesthesia and inhalation anaesthesia. Eur J Cardiothorac Surg.

[5] Beverstock J, Park T, Alston RP, Song CCA, Claxton A, Sharkey T (2021). A comparison of volatile anesthesia and total intravenous anesthesia (TIVA) effects on outcome from cardiac surgery:a systematic review and meta-analysis. J Cardiothorac Vasc Anesth.

[6] Edwards ZE, Kelliher LJ (2020). Propofol-TIVA versus inhalational anesthesia for cancer surgery. Dig Med Res.

[7] Lenartova K, Sear C, Al Hannoush N, Moravcova S, Alhalabi W, Marczin N (2023). Total intravenous anaesthesia versus inhalational anaesthesia and influence on acute postoperative pain after thoracic surgery. J Cardiothorac Vasc Anesth.

[8] Niu Z, Gao X, Shi Z, Liu T, Wang M, Guo L (2021). Effect of total intravenous anesthesia or inhalation anesthesia on postoperative quality of recovery in patients undergoing total laparoscopic hysterectomy:A randomized controlled trial. J Clin Anesth.

[9] Jo JY, Kim YJ, Choi SS, Park J, Park H, Hahm KD (2021). A prospective randomized comparison of postoperative pain and complications after thyroidectomy under different anesthetic techniques:volatile anesthesia versus total intravenous anesthesia. Pain Res Manag.

[10] Wong SSC, Choi SW, Lee Y, Irwin MG, Cheung CW (2018). The analgesic effects of intraoperative total intravenous anesthesia (TIVA) with propofol versus sevoflurane after colorectal surgery, Medicine (Baltimore).

[11] Ramirez MF, Gan TJ (2023). Total intravenous anesthesia versus inhalation anesthesia:how do outcomes compare?. Curr Opin Anaesthesiol.

[12] Trapani G, Altomare C, Sanna E, Biggio G, Liso G (2000). Propofol in anesthesia. Mechanism of action, structure-activity relationships, and drug delivery. Curr Med Chem.

[13] Zhang Y, Zhao L, Lv L, Li S (2022). Retrospective analysis of remifentanil combined with dexmedetomidine intravenous anesthesia combined with brachial plexus block on shoulder arthroscopic surgery in elderly patients. Pak J Med Sci.

[14] Chen RM, Chen TG, Chen TL, Lin LL, Chang CC, Chang HC (2005). Anti-inflammatory and antioxidative effects of propofol on lipopolysaccharide-activated macrophages. Ann N Y Acad Sci.

[15] Li M, Mei W, Wang P, Yu Y, Qian W, Zhang Z (2012). Propofol reduces early post-operative pain after gynecological laparoscopy. Acta Anaesthesiol Scand.

[16] Pokkinen S, Yli-Hankala A, Kalliomäki ML (2014). The effects of propofol vs. sevoflurane on post-operative pain and need of opioid. Acta Anaesthesiol Scand.

[17] Lee BH, Park JO, Suk KS, Kim TH, Lee HM, Park MS (2013). Pre-emptive and multi-modal perioperative pain management may improve quality of life in patients undergoing spinal surgery. Pain Physician.

[18] Katz J, Seltzer Z (2009). Transition from acute to chronic postsurgical pain:risk factors and protective factors. E Expert Rev Neurother.

